# Voids Development in Metals: Numerical Modelling

**DOI:** 10.3390/ma16144998

**Published:** 2023-07-14

**Authors:** Wiktor Wciślik, Sebastian Lipiec

**Affiliations:** 1 Faculty of Civil Engineering and Architecture, Kielce University of Technology, 25-314 Kielce, Poland; 2 Faculty of Mechatronics and Mechanical Engineering, Kielce University of Technology, 25-314 Kielce, Poland; slipiec@tu.kielce.pl

**Keywords:** porous ductile material, numerical modelling, void nucleation, growth, coalescence, second phase particle, particle cracking, debonding

## Abstract

The article is a continuation of two previous review papers on the fracture mechanism of structural metals through the nucleation, growth and coalescence of voids. In the present paper, the literature on the numerical modelling of void nucleation and development has been reviewed. The scope of the work does not include porous material models and their numerical implementation. As part of the discussion on void initiation, nucleation around second phase particles and nucleation as an effect of the discontinuity of the crystal structure were discussed separately. The basic void cell models, finite element method (FEM) models of periodically distributed particles/voids and models based on the results of the observations of the actual microstructure of materials have been characterised. Basic issues related to the application of the cohesive approach in void nucleation modelling have been considered. A separate issue is the characteristics of atomistic simulations and peridynamic modelling, which have been developed in recent years. Numerical approaches to modelling the growth and coalescence of voids are described, with particular emphasis on the influence of the stress state and strain localisation. Basic conclusions from the simulation are presented, pointing to the contribution of FEM modelling to the understanding of microstructural phenomena leading to ductile fracture.

## 1. Introduction

In recent years, significant progress in understanding the phenomenon of the ductile fracture of metals associated with the growth of voids has been observed. Modern research techniques allow for the three-dimensional observation of the development of voids in a wide range of deformations, up to failure [1,2,3,4,5]. However, it should be remembered that the discovery of these phenomena is the basis of the next stage, which is the computer modelling of damage, taking into account the mechanisms that occur at the level of the material’s microstructure. Models of ductile materials with micro-damages, developed over the last few decades, are widely known [6,7,8,9,10,11,12,13]. However, porous plasticity models are based on numerous assumptions, which are not always confirmed by observations. For example, it is common to idealise the shape of the voids (usually assuming their spherical shape), while in fact the shapes are irregular (e.g., voids nucleating in the area of elongated bands of MnS inclusions). In addition, it is most often assumed that the shape of the voids remains unchanged over the entire deformation range, which also contradicts experimental observations. The current approach to modelling includes either a single void or only small groups of them, which does not allow the complete incorporation of their interactions and only partially reflects the statistical nature of the entire process. Although material models that take into account the diverse geometry of voids have been proposed in recent years [14,15,16], the problem has still not been fully solved.

The further development of micromechanical material models and taking into account the observed phenomena are therefore conditioned by the exact characterisation of the nucleation, growth and coalescence of voids through analytical and numerical models. However, this is probably the least developed area related to this problem. The current models of nucleation, growth and void coalescence often provide results that are not entirely consistent with the experiment; therefore, their comprehensive development is crucial.

This review article presents the current state of knowledge relating to the numerical modelling of the nucleation, growth and coalescence of voids in metal alloys for technical applications. It is a continuation of the subject discussed in [17,18]. Thus, the paper [17] presents general information on the phenomenon of the formation and development of voids in metals, together with a discussion of the basic, classical analytical models. In turn, in [18], the results of the experimental research and in situ observation of the development of voids were characterised, including the use of modern research methods, such as microtomography. The present paper complements the previous ones in terms of modelling the microstructural phenomena, not the material. As before, the scope of the paper is limited to metallic materials, loaded statically, monotonically and at room temperature. Therefore, non-metals (for example, polymers in which similar phenomena are observed), as well as the influence of element operating conditions (strain rate, temperature, thermal cycling [19] etc.) on the fracture behaviour, are not considered here.

## 2. Void Nucleation

### 2.1. Void Nucleation at Second Phase Particles

It is commonly believed [20], that the nucleation of voids in metals for technical applications is most often the result of second phase particles cracking or the particle/matrix separation. Starting in the 1960s, numerous analytical models of nucleation have been developed, based on stress, strain or energy criteria. An overview of the analytical models can be found in [17].

However, due to the engineering importance of the problem, many papers have been devoted to the finite element method (FEM) modelling of these phenomena. In order to fully describe the void nucleation, factors such as the material, size, shape of particles, their clustering and state of stress should be taken into account. The temperature, strain rate and pre-strain are also crucial [21]. The numerical estimation of stress and strain in the particle and the surrounding matrix makes it possible to formulate the criteria for void nucleation through particle cracking or its separation.

**Figure 1:**
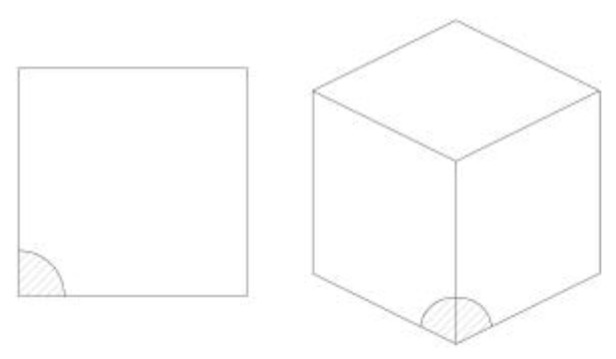
Exemplary 2D and 3D unit cell computational models.

The basic type of numerical model of a void or a nucleating particle is a two- or three-dimensional unit cell model [22]. The 2D model can represent a flat piece of material with a cylindrical particle/void placed in it, or it can be an axisymmetric model in which one of the symmetry axes is also the axis of rotation; therefore, the whole model represents a rotationally symmetric particle in a cylindrical matrix. A separate category is 3D models (usually a cubic matrix with a particle/void located at the intersection of the axes of symmetry). Generally, the overall behaviour of the unit cell results from competition between the strain hardening and softening induced by the growing void [23]. The most common configurations are illustrated in Figure 1.

The choice of the type of model (2D, axisymmetric, 3D) depends on the expected results, the shape of the particle and the type of applied loads. The use of a 3D model is therefore necessary for particles with complex shapes and in cases where an analysis of the effect of the Lode parameter is required (especially a combination of tension and shear) [23].

Although the determination of the particles geometry (size, shape) may be based on the results of microstructural observations, simplified shapes (usually spherical or elliptical) are most often used for simulation. The main difficulty, however, is the proper identification of the material parameters of the matrix and particles, as well as the properties of their interface. Basically, the matrix is modelled using the J2 flow theory, although, depending on the purpose and scope of modelling, more complex descriptions are used, taking into account plastic anisotropy [24], rate dependency [25] and others. Depending on the size of the modelled particle, matrix stress–strain relationships should be determined at the dislocation (small particles) or continuum mechanical scale (larger particles) [26]. In the latter case, the properties of the matrix are usually identified with the properties of the material at the macroscopic level. Another serious problem is the determination of the mechanical properties of the microscopic second phase particles. Measuring these parameters, although technically possible in the present day [27,28], is complicated, time-consuming and expensive. The situation is slightly improved by the fact that the particles of the second phase are most often modelled as elastic or brittle, which eliminates the need for the tedious and difficult determination of plastic properties.

**Figure 2:**
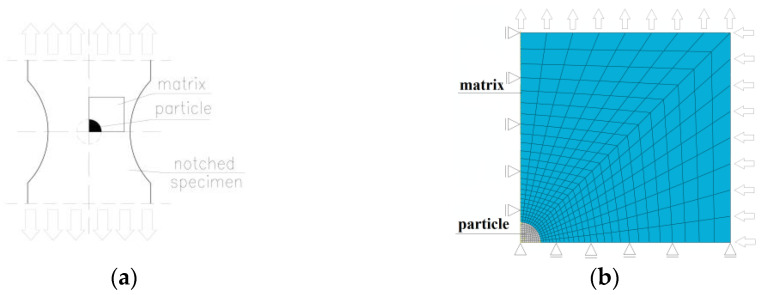
(**a**) Boundary conditions and (**b**) Numerical model of inclusion for simulation of void nucleation, from [29].

Examples of the use of a cell model to simulate the initiation of voids around second phase particles in S355 steel are discussed in [29,30]. It was assumed that the analysed particle was extracted from the ring-notched specimen, which implied a specific state of stress to which the particle was subjected (Figure 2a).

Axisymmetric models (Figure 2b) were used, in which the particle geometry (shape, size) was adopted on the basis of the results of microstructure observations. The matrix was modelled as an elastic-plastic material with non-linear hardening. Fe_3_C and MnS inclusions were considered, assuming the elastic material characteristics The value of the Young’s modulus and tensile strength were adopted according to [31].

Particular attention was paid to the mechanism of inclusion/matrix debonding. It was assumed that separation occurs when the radial stress (determined according to [32]) reaches a critical value, characteristic for the inclusion material, according to [32,33,34]. Depending on the type of the considered particle and the mechanism of nucleation (cracking or separation), the critical values of nucleation strain—ranging between around 1 and nearly 30%—were obtained, with higher values observed in the case of matrix/particle separation. Additionally, within the framework of the approach described above, the effect of the stress state triaxiality (*η*) on the nucleation strain was analysed, finding that, in the analysed range, the strain value decreased with the increasing triaxiality.

**Figure 3:**
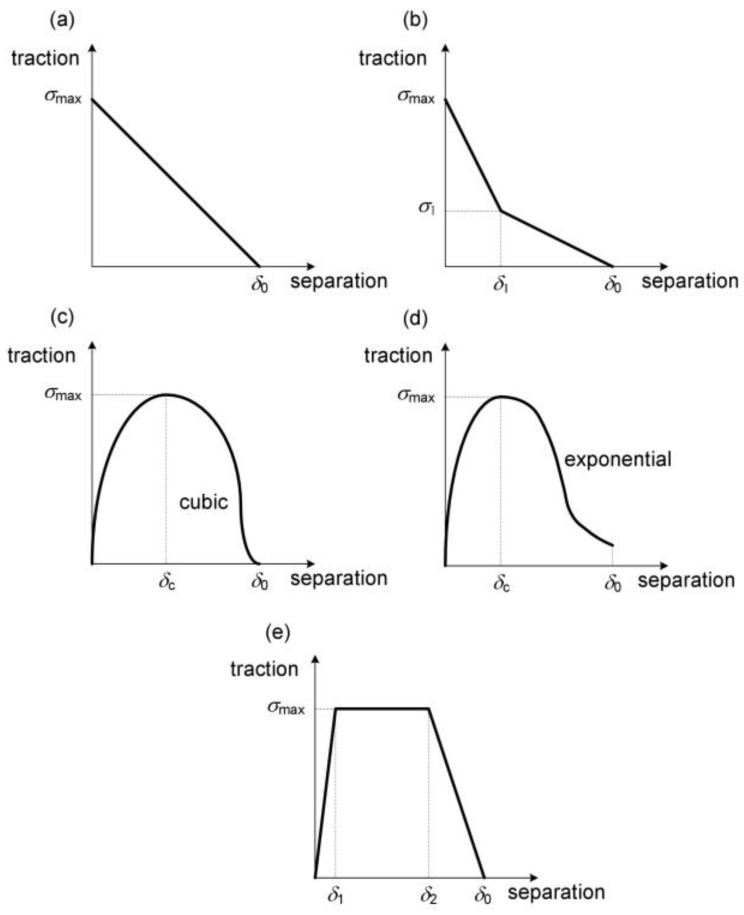
Traction-separation laws (TSLs) proposed in the literature: (**a**) Hillerborg [37]; (**b**) Bažant [38]; (**c**) Needleman [39]; (**d**) Needleman [40]; (**e**) Tvergaard and Hutchinson [41], reproduced from [42].

When modelling the matrix/particle separation, it is of particular importance to define the interface properties [35,36]. A convenient solution in this case is the cohesive approach, in which the relationship between the traction (stress) and separation (elongation) is defined. The concept of cohesive models assumes that when the critical separation *δ*_0_ is reached, the traction drops to 0, which is equivalent to the initiation of a void at the interface [17]. Examples of the traction-separation laws (TSLs) described in the literature are presented in Figure 3.

Moreover, the cohesive laws are defined separately for the normal direction and the directions tangent to the interface surface [43].

As before, the key issue for the quality of the results is the correct identification of the cohesive parameters. This problem will be addressed later in this work.

One of the early comprehensive numerical simulations of void nucleation in the vicinity of a particle, in terms of the cohesive approach, was described by Needleman in [39]. The boundary value problem of rigid spherical inclusion in an isotropically hardening elastic viscoplastic matrix was analysed. Particle/matrix separation was modelled using a cohesive interface model. Importantly, both the problem of void initiation and the subsequent crack propagation until complete decohesion at the interface were analysed. The concept of the characteristic length was introduced, indicating that, depending on the relation of this value to the radius of the particle, the separation is “brittle” or “ductile”. The first case concerns large particles (relative to the characteristic length), while the second one is obtained for smaller particles.

The performed analysis revealed that the void nucleation strain is strongly dependent on the stress state triaxiality, with an increase in triaxiality being accompanied by a decrease in strain. Similarly, it was observed that in the analysed range, larger particles required lower strain to initiate the void. However, this effect reduces and finally disappears in the case of particles with a diameter of several micrometres and larger [44].

The author of [39] also pointed out that, in the case of the numerical evaluation of the void nucleation strain, interpretation ambiguities appear because the moment of nucleation can be considered as both the initiation of particle/matrix interface cracking or complete particle separation.

At the same time, the author indicated some limitations to the applied cohesive model, stating that it is appropriate for relatively weak matrix/particle interfaces, where the stress gradients are relatively small. Finally, the model is a continuum one, which excludes the possibility of taking into account the effect of dislocation.

This approach was developed by Charles et al. [45], who proposed a cohesive model in which the maximum traction is dependent on local plastic deformation, which in turn is related to dislocation accumulation. This solution was used in modelling the competition between particle fracture and debonding in metal matrix composites. Namely, it was found that particle debonding occurred with a soft matrix, while particle fracture dominated with a hard matrix. These conclusions were consistent with the microstructural observations.

Yu [46], using a 3D unit cell model with a cohesive matrix/particle interface, analysed the influence of the stress state on void nucleation and growth. The results obtained for the unit cell with a particle and with a pure void were compared. It was found that with the same triaxiality, the presence of the particle increased the cell load-carrying capacity and reduced the void growth. Moreover, it was noticed that even with constant triaxiality, a change in the Lode parameter/angle (*L*) caused a change in the nucleation site of the interfacial crack and a change in the direction of its propagation. The effect of the cohesive parameters on the simulation results was also assessed by changing the ratios of shear to normal separation and the shear to normal cohesive energy. However, these modifications, in the assumed range, had no effect on either the macro equivalent stress or the relative void volume fraction.

**Figure 4:**
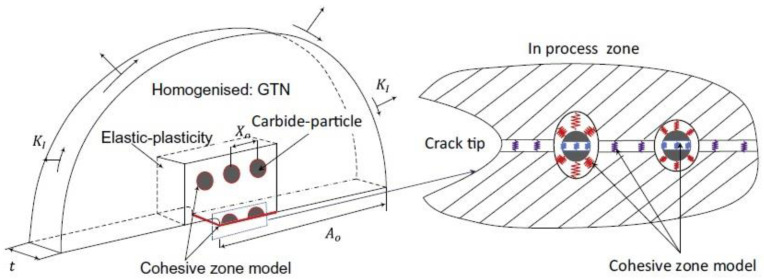
Scheme of modelling 3 damage initiation mechanisms at the crack tip: particle fracture, particle debonding, cleavage of ferrite matrix, reprinted with permission from Ref. [47]. 2023, Elsevier.

Giang et al. [47] presented a more comprehensive analysis of failure initiation around the particles deployed in front of the crack (Figure 4). As before, cohesive models were used, this time adding to the two mechanisms mentioned above (particle cracking and debonding) a third one, related to the cleavage fracture of the ferritic matrix in steel. Inside the process zone, an elastic-plastic matrix model was used, while outside it, the heterogeneity of the material (the presence of voids) was taken into account in a homogenous way using the Gurson-Tvergaard-Needleman (GTN) material model. Such a comprehensive approach made it possible to describe the complex ductile-brittle transition processes that were observed experimentally. Moreover, the model being constructed in this way made it possible to determine the effect of the particle strength on the fracture toughness.

A similar analysis (in terms of matrix/particle separation) was carried out by Testa et al. [48]. The authors analysed the effect of the stress state triaxiality on the critical strain of void nucleation in α-iron. For this purpose, a unit-cell model of an elastic spherical particle embedded in an elastic-plastic matrix was used. A simulation of the decohesion of both phases was carried out using a cohesive model. The key problem in identifying the model parameters was solved by associating them with macroscopic material properties. The separation energy was recognised through the critical value of the energy release coefficient *G_c_*, while for the cohesive opening displacement *v_m_*, a simple relationship was proposed:

(1) $$v_{m}=\frac{G_{c}}{m^{\prime }\;\sigma_{y}}$$

where: *v_m_*—cohesive opening displacement, *G_c_*—the critical value of the energy release coefficient, *σ_y_*—yield stress, *m*′—plastic constraint factor.

Based on the simulation results, it was found that the critical strain of the void nucleation decreases exponentially with the increase in triaxiality, which confirms the results in [29,30,39].

In turn, Andersen et al. [49] attempted to numerically determine the influence of the morphology of particles acting as sites of void nucleation on the global cohesive parameters describing steady-state ductile plate tearing. On the basis of the simulation, it was found that the inhomogeneity of the material resulting from the nucleation of voids strongly affects the location of damage, and thus the cohesive energy decreases with the increase in the number and size of particles. On the other hand, the peak force in the cohesive traction-separation law turned out to be insensitive to changes in the morphology of randomly distributed particles.

When modelling the phenomenon of particle and matrix separation, it is usually assumed that in a non-deformed material, the interface connection is perfect, while experimental observations indicate nanoscale defects between both phases. Recently, Liu et al. [50], in the framework of a numerical approach, proposed a stress-inclusion debonding criterion, taking into account the nanoscale damage of the interface. As previously mentioned, in contrast to other solutions, the proposed criterion includes the influence of particle size on the value of deformation for void initiation. This relationship is consistent with the results of the experiments and observations of the microstructure of materials, where it was found that the value of the critical strain increases with the decreasing particle size. Moreover, the authors [50] compared the simulation results using the stress and the energy criterion, confirming the common belief that the energy criterion is met at low strain values and is not sufficient to initiate the void [17].

Another modern and promising technique is atomistic simulation. In this case, the material’s behaviour is described at the atomic level, which overcomes many limitations of traditional local and non-local models. It is a flexible and universal approach, as it is based on the definition of the type and position of individual atoms, as well as the model of interaction between them. Atomistic simulation therefore allows for the simultaneous modelling of various failure mechanisms, in which crack propagation is understood as the breaking of successive interatomic bonds. A distinction is made here between models based on force field methods (operating on Newtonian mechanics) and quantum mechanics-based models, in which the interaction between atoms is primarily the result of the interaction of electrons. This makes it possible to model a wide range of material properties, not only mechanical ones, for example, magnetic issues, thermal and electrical conductivity and all other properties that result directly from the principles of quantum mechanics.

Models using force field methods are used to analyse the mechanical energy, forces and stresses in a material, as the forces acting on a single atom are dependent on the type and position of other atoms in its vicinity. This approach is more computationally efficient; therefore, it is possible to analyse larger material volumes than in the quantum mechanics approach. In the mechanics of materials, the most frequently used type of force fields method is molecular dynamics (MD) simulation, which uses Newton’s second law (the acceleration of an atom is determined as the quotient of the force acting on it and its mass). The combination of this type of simulation with machine learning methods offers particularly great opportunities to develop new complex forcefields.

Despite the versatility and many possibilities offered by atomistic simulation, its application in engineering issues brings some difficulties. Firstly, the analysis of fracture issues requires the construction of extensive models, which is a highly computationally demanding task. Secondly, the discrepancy between time- and length-scales in atomic and engineering issues also causes problems. For example, in MD, the maximum time step is dictated by the frequency of thermal vibrations (most often given in femtoseconds), while for engineering problems, seconds are generally used. To overcome this problem, some multiscale methods have been developed [51].

The atomistic simulation approach is particularly useful in the analysis of the initial phases of void nucleation and the assessment of the accompanying physical phenomena, the experimental capture of which, due to technical difficulties, is troublesome or sometimes even impossible. For instance, Zhao et al. [52], using molecular dynamics (MD) analysis, simulated the separation of a spherical Al_2_Cu *θ* precipitate in an aluminium matrix. Reaching the atomic level made it possible to distinguish three stages of the early phase of decohesion, namely: a nucleus of excess free volume at the phases’ interface, nuclei growth in the absence of dislocation activity and the emission of Shockley partial dislocations leading to fast microcrack development.

Similarly, Lucchetta et al. [53] assessed the strength properties of particulate-reinforced nanocomposite using molecular dynamics analysis of a cubic cell comprised of an aluminium matrix and a spherical nickel nanoparticle. The authors found that the effective strength increases with the inclusion size reduction at a constant reinforcement volume fraction.

In order to fully understand the process of nucleation and the development of voids, it is necessary to consider their interaction. This applies, among others, to nucleation within particles grouped close to each other in clusters. The development of voids in clustered structures is slightly different than in isolated cells with a single particle. This difference results not only from the non-spherical shape of the cluster, but also from the localisation of stress and strain in the ligaments between voids. The local anisotropy of the material introduced by the presence of the cluster affects all stages of the ductile fracture.

One of the better-known papers on the numerical analysis of the effect of voids clustering on their development is [54]. Using unit cell models, three types of void arrangements were analysed: cubic, body centred and hexagonal. Unit cells containing initially spherical voids, arranged periodically, were subjected to a complex state of stress, with the prescribed triaxiality kept constant throughout the loading history. It was found that the arrangement of the voids only slightly affects the deformation behaviour. On the other hand, by positioning the arrangements in the order of hexagonal, cubic and body centred array, an increase in hardening, a higher failure load at larger strains and a decrease in the evolution of void volume fraction were observed.

Thomson et al. [55] analysed the effect of the particle cluster orientation on void nucleation and development. Based on three-dimensional FEM models, several types of particle distribution were considered: linear, planar and quasi-spherical. The effect of the particles’ concentration was incorporated by introducing the normalised distance between them. The phenomenon of matrix/particle separation was considered, without a particle cracking mechanism. It was found that in the early stage of deformation (before nucleation), clusters oriented along the direction of the maximum principal strains experience the greatest localisation of plastic strains, which results in the early nucleation of voids. Later, after the formation of a void, the trend reverses, i.e., the largest deformation is observed around the clusters oriented perpendicularly to the major strain axis, which results in the most intensive development of the defect. Similar results were described in [56], while indicating that complex clusters of particles/voids subjected to multiaxial deformation states after the initialisation of the first voids are subjected to additional constraints; that is, before a significant growth of the already initiated voids starts, new voids must be formed on all of the particles within the cluster.

Although many papers on particle clustering have been published in the literature, they certainly do not entirely solve the problem as they deal with idealised structures (in terms of particle shapes and distribution) that usually have no counterpart in real materials. Some progress in this regard has been made in recent years due to the development of finite element modelling (FEM) and remeshing techniques associated with the modern tools of 3D structures mapping.

For example, in the works of Roux and Shakoor et al. [57,58,59], a thorough analysis of the development of voids was carried out for larger 2D and 3D structures, also taking into account the influence of second phase particles.

In [57], a 2D numerical model of void nucleation and growth was developed for large deformations. The change in the cluster geometry at high strains was taken into account using the level-set (LS) method [60] and advanced remeshing techniques. Both the particles’ cracking mechanism and the particles/matrix separation were taken into account based on the stress criteria.

**Figure 5:**
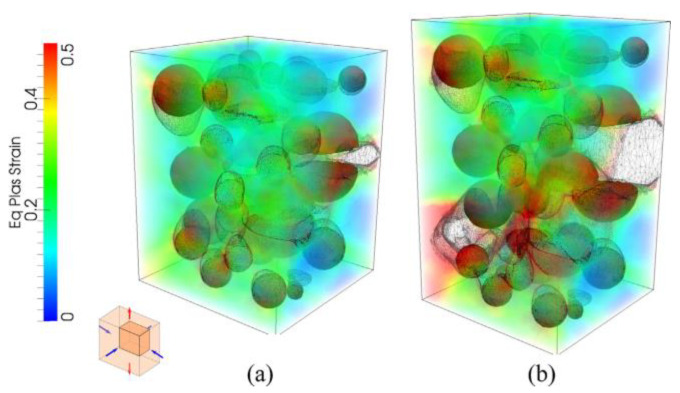
Microstructure and strain maps of RVE: (**a**) At global RVE elongation 25%; (**b**) At 50%, reprinted with permission from Ref. [59]. 2023, Elsevier.

The above concept was developed for a 3D case in [59] for the fracture analysis of an aluminium alloy with a large (20%) initial volume fraction of zirconia-silica (ZS) particles. The representative volume element (RVE) model with particles of an initially circular shape, various sizes and irregular distribution was used for the simulation. As previously mentioned, the use of modern remeshing techniques made it possible to analyse the material in a wide range of deformations, including high values of plastic strain. Both the matrix/particle separation mechanism and particle fracture were analysed, with the latter mechanism being dominant in this case. In addition, the issue of particle pre-fragmentation and particle-free material (matrix + voids) were taken into account. Based on the simulation results, it was found that the nucleation of voids leads to the localisation of plastic deformation, which in turn accelerates the coalescence of voids and significantly reduces the ductility. An exemplary RVE microstructure with deformation maps is presented in Figure 5.

Shabrov and Needleman [61] performed a numerical evaluation of the strain at the onset of void nucleation around inclusion, taking into account the particles’ clustering. Double periodic arrays of elastic inclusions placed in an elastic-viscoplastic matrix with an isotropic hardening were analysed. It was assumed that the elastic properties of the particle and the matrix are the same, which limits the scope of considerations to only some types of particles (mainly carbides in structural steels). There were nine different variants of particle distribution (uniform and random). In each of the analysed cases, all of the particles were the same size. The load was applied as biaxial tensile stress under plane strain conditions. The contact between the particles and the matrix was modelled using the cohesive constitutive relation [39]. The authors found that under low triaxiality conditions (below about 0.4), nucleation of the voids occurred at relatively high strains (up to about 12%), with higher strain values observed for regular distributions. With the increase in triaxiality, the nucleation strain decreased. In this case, the size of the particles had a more significant effect on the nucleation strain than their distribution (greater values of strain were observed for small particles). Moreover, based on the obtained results, the function of the effective separation stress, linearly dependent on the hydrostatic stress, was formulated.

Butcher and Chen [62] described an example of a strain-controlled void nucleation simulation in an AA5182 aluminium alloy based on the damage percolation model. Using the digital imaging technique, the geometric parameters and distribution of the particles in the bulk material were determined. Then, on this basis, a numerical model was prepared, considering the actual geometry of the particles, and thus allowing the local character of crack initiation to be captured. Three particle fields were subjected to different levels of uniaxial and biaxial stretching.

In order to determine the onset of void initiation, a phenomenological nucleation model was proposed, which considered the particles size, their surface area and the influence of the stress state. The latter factor was quantified by the stress triaxiality, defined as the ratio of the mean to effective stress (taking into account the influence of hydrostatic pressure). However, the stress triaxiality itself does not fully describe the prevailing state of stress because it does not take into account the shear stress. Therefore, the Lode parameter was included in the void nucleation model in order to deal with the effect of the shear stress and the third invariant of the stress tensor [63,64].

The elliptical shape of the particles and their variant configurations (along and across the rolling direction) were assumed. Particles composed of different elements (namely Fe- and Mn-based particles) were not distinguished. The effect of the type of nucleation mechanism (particle cracking/separation) was not analysed. It was assumed that in the initial state, the material is free from damage. In the next step, the model parameters were calibrated by comparing the simulation results with the experimental data according to [65]. After calibration, the forming limit curves (diagrams of minor versus major strain) obtained with using the proposed model were characterised by a very good agreement with the experiment described in [65].

Another issue that is relatively often analysed in the literature is the determination of the critical stress necessary to initiate the void. For example, Shabrov et al. [66] used a mixed experimental–numerical method to determine the critical fracture stress of TiN particles in titanium-modified 4330 steel. In the first stage, tensile tests were performed on notched specimens subjected to a complex state of stress. The tests were stopped at various stages and microstructural analysis of the deformed material was performed. Particular attention was paid to a thorough inventory of the TiN particles and the identification of areas where they had cracked. In the next stage, the map of cracked particles was superimposed onto the numerically determined stress maps, thanks to which it was possible to determine the stress criterion of cracking TiN particles at the level of approximately 2.3–2.4 GPa. In addition, various analytical criteria for void nucleation were verified, indicating that the criterion based on the weighted sum of hydrostatic tension and effective stress provides the best agreement of results.

### 2.2. The Role of Grain Boundaries

The voids’ initiators can also be grain boundaries, especially the points of the grains’ triple junctions. In recent years, due to the constant development of computational methods, simulations have allowed for a better understanding of these phenomena.

Pardoen et al. [67] analysed the process of void development as a result of competition between intergranular and intragranular fractures in aluminium alloy. A bilayer FEM model of the elementary cell was prepared, in which the first layer represented grain behaviour, where, due to precipitation, high yield stress and low hardening exponent were observed. The second layer represented the precipitation free zone (PFZ) in the vicinity of the grain boundary, characterised by opposing characteristics, i.e., low yield stress and high strain hardening capacity. The failure mechanism of the grain boundaries (in the vicinity of PFZ) was favoured by a high value of triaxiality coefficient, a small value of void spacing to void diameter ratio and a high value of PFZ thickness to void spacing ratio.

Recently, Sui et al. [68] developed and numerically validated a model of the nucleation of voids on grain boundaries under dynamic loading, taking into account the interface incompatibility. The critical nucleation stress was dependent on the void embryo size, the load angle and the grain boundary (GB) characteristics (incompatibility of mechanical properties across the GB).

The modelling of failure initiation at the microstructural level in multiphase materials often leads to numerical errors and instabilities. To reduce and overcome this problem, peridynamic (PD) modelling [69] has been proposed, in which no spatial derivatives are employed in the equations of motion. As a result, the numerical algorithm is more stable. The PD method is therefore a useful tool for modelling material discontinuities and progressive failure without the need to introduce additional damage models. For example, Ahmadi et al. [70] used the PD technique to model void nucleation in dual phase DP600 steel. A two-dimensional model of the ferrite and martensite phases was made on the basis of the actual microstructure, determined through scanning electron microscopy (SEM) observations. In addition, a method for determining the strength parameters of the ferrite/martensite interface, based on the parameters of individual phases, was proposed. Then, an analysis of the material subjected to static tension was carried out. The simulation allowed for the successful prediction of damage initiation and propagation. Voids were initiated through the cracking of martensite grains and interface separation.

The aforementioned atomistic simulation approach is also used to analyse void nucleation at grain boundaries [71].

**Figure 6:**
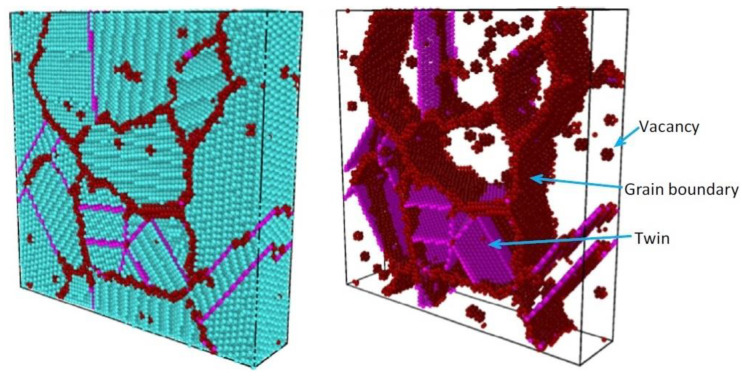
Representative volume element (RVE) for molecular dynamics simulation of nano-void nucleation in nanocrystalline aluminium, reprinted with permission from Ref. [72]. 2023, Elsevier.

For example, the authors of [72] used this method to assess the nucleation conditions of nano-voids on aluminium grain boundaries under uniaxial, plane strain and equi-biaxial loading conditions. A numerical model of the representative volume element (RVE), composed of 100,000 atoms with a perfect face centred cubic (FCC) structure, was developed. The atomic interaction was described in terms of its EAM potentials [73]. The material deformation at a speed of 10^9^ s at room temperature was simulated. The structure of the analysed RVE is presented in Figure 6.

**Figure 7:**
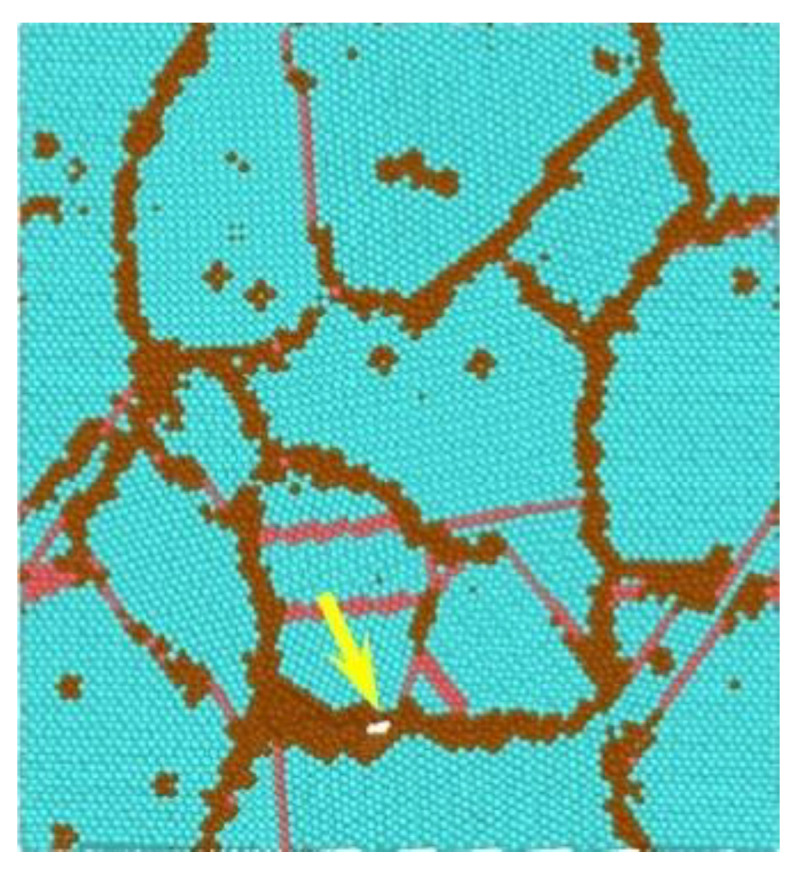
Nano-void nucleation predicted by molecular dynamics simulation under plane strain conditions, reprinted with permission from Ref. [72]. 2023, Elsevier.

The simulation results showed that, regardless of the stress state, void nucleation occurred at the grain boundaries, at triple points, with strains ranging between around 9 and 18%, with the lowest strain values recorded for plane strain. Then, the initiated defect propagated along the grain boundaries, leading to failure, which occurred at strains beginning at almost 12% (plane strain) to nearly 30%. The intergranular properties of the material failure was verified by the previous observations in FCC materials [74,75]. An example of nano-void visualisation in plane strain conditions is presented in Figure 7.

### 2.3. Other Issues

The simulation of nucleation and the development of voids in real technical materials, not only parameterised ones, brings additional difficulties, primarily the need to identify the material parameters, which is crucial for the quality of the obtained results. These parameters may include the stress–strain relationship of the matrix and particles, the traction-separation law for the particle/matrix interface, etc.

The strength properties of the matrix are most often determined macroscopically, using a uniaxial tension test. This approach is a certain simplification because the obtained results apply to all phases of the material, and not only to the matrix itself. This is of particular importance in materials with a high particle content, e.g., metal matrix composites.

A solution to this problem was proposed by Buljac et al. [76]; however, this required complex experimental studies related to FEM simulations. The first step of the procedure included the analysis of the microstructure of the commercial nodular graphite cast iron sample, using X-ray microtomography, to obtain 3D images of the material. Further, during the tensile test, the Digital Volume Correlation (DVC) technique was employed to capture displacement fields in the material subjected to deformation. In the next stage, an FE simulation was carried out by considering the actual microstructure of the material (registered by X-ray tomography) and displacement fields obtained using DVC. Subsequently, on this basis, the plastic behaviour of the ferritic matrix was calibrated.

Another problem discussed in the literature is the analysis of the development of voids in front of crack. The authors of the paper [77] numerically analysed the interaction of a crack and a cluster of voids located ahead of a pre-existing crack tip. By analysing the two-dimensional plane strain model, two different mechanisms of crack growth were found, both involving the development of initially circular voids. With a sufficiently low initial fraction of voids, crack propagation followed the mechanism of the sequential, independent growth of individual voids. In the case of larger initial fractions, several voids ahead of the tip increased simultaneously, giving rise to crack development. The transition between the two mechanisms took place in a wide range of initial fractions, i.e., from about 0.001 to 0.005. This is a typical range for many metal alloys for technical applications, which does not allow for an unambiguous indication of the mechanism typical for structural materials.

In recent years, Liu et al. [78] conducted similar analysis, considering not only the voids, but also two populations of void nucleating particles. A three-dimensional model of the element, loaded according to method I (tearing), was developed. Large particles (nucleating voids with small strains) were modelled discretely, while small particles (requiring large strains for nucleation) were distributed homogeneously. The size, volume fraction of the particles and the distance between them defined the microstructurally based length scales. Contrary to [77], the analysis of the random distribution of particles in a ductile matrix did not allow the authors to clearly state whether the crack propagation is the result of void by void growth or simultaneous multiple void development. However, it was observed that the phenomenon of the localisation of plastic deformations is favoured by their large particle size and their large volume fraction. An interesting aspect of the work [78] is also the coupling between the microstructural parameters of the material and fracture toughness. It was found, among others, that in the case of small particles, increasing the distance between them has a significant impact on the fracture toughness. This effect was, in turn, smaller in the material containing large particles.

In the literature, the issue of void nucleation inhomogeneity, related to the statistical nature of nucleation parameters (particle and interface strength, nucleation strain value, etc.), is relatively rarely raised. This inhomogeneity affects the local state of stress and the course of void development. Recently, Chen [79] compared the evolution of the stress triaxiality and the Lode parameter in a sheet subjected to simple shear deformation for homogenous and heterogenous void nucleation. Additionally, two variants of the initial material microstructure were considered: unvoided and with pre-existing voids placed in the sheet centre. In the first case, a slight effect of the nucleation heterogeneity on the triaxiality and Lode parameter was observed. In the second case, the state of stress was to a large extent determined by the presence of the void, and therefore further homogenous and heterogenous nucleations were indistinguishable. This leads to the conclusion that at advanced levels of deformation (when the material already contains a significant number of voids), nucleation heterogeneity is insignificant.

## 3. Numerical Analyses of the Mechanism of Growth and Coalescence of Voids

In the numerical analysis of the growth process and the coalescence of voids, the predominant method is the use of the elementary cell in the calculations, as an example of the use of the RVE model (representative volume element) (the subject of the elementary cell has been discussed in many scientific studies, including the following [24,80,81,82,83,84]). In the following section, a literature review and selected results obtained by carrying out numerical calculations using the finite element method will be presented. The considerations will be presented in such a way as to present the influence of individual parameters on the growth process and the coalescence of voids occurring in the material. However, it should be borne in mind that the numerical models are inextricably linked to the experimental observations. When analysing the literature, it can be seen that there is now a fairly general consensus on the physical modelling of void growth processes. This is also reflected in the numerical verification of the phenomenon. This is, among other things, is due to the mechanics of the process itself. The situation is more complicated when it comes to the experimental and numerical description of the void coalescence process. This is due, among other things, to the complexity and dynamics of the process, as well as the difficulty in numerically describing the phenomenon. Several mechanisms for the realisation of void coalescence in a material can be found in studies by researchers: the complete breaking of the ligament between voids; instability of void growth or instability of cavitation; coalescence between one large void and several small voids nucleating on a second population of inclusions, leading to the formation of a ring of damaged material accelerating the coalescence between larger voids; bonding of voids through plastic shear localisation (formation of a sheet of voids) or the occurrence of necking between voids interrupted by plastic stretching localisation (a phenomenon related to the unstable growth of the second population of voids) [85,86,87,88].

### 3.1. Shape and Configurations of Voids

**Figure 8:**
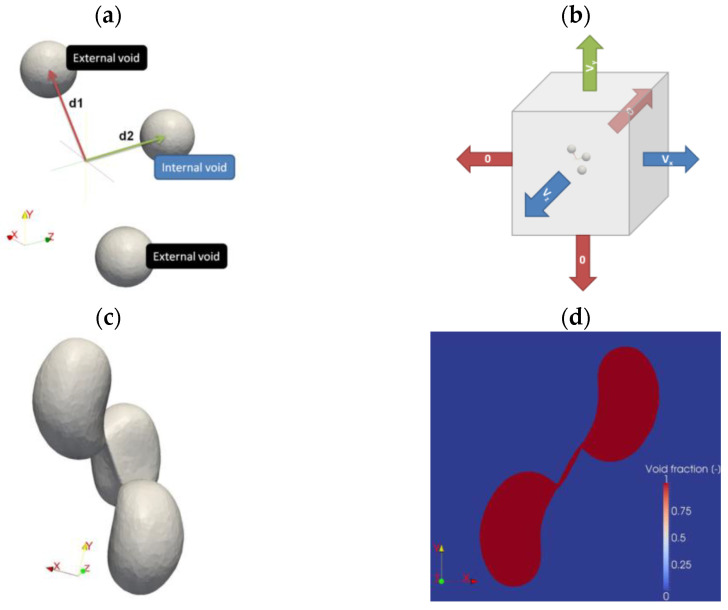
The assumption of the model was made: (**a**) characteristic dimensions of the void distribution, (**b**) scheme of boundary conditions, (**c**,**d**) results obtained after the coalescence process (equivalent strain 0.13, *d*_1_ = 1, *d*_2_ = 1), reprinted with permission from Ref. [89], 2023, Elsevier.

In order to assess the influence of the size and distribution of voids relative to each other on the growth and coalescence of voids in the numerical analysis programme, a three-dimensional cluster with three voids was adopted as the model in the work of Trejo-Navas et al. [89]. The voids in the initial state were characterised by their spherical shape. The characteristic dimensions of the system and the model adopted for the analyses are shown schematically in Figure 8. The adopted methodology for modelling the elements of the material microstructure—the clusters of voids—enables the analysis of non-periodic clusters, as well of the use of elements with non-idealised shapes in the model while controlling the levels of the parameters of the stress state (stress triaxiality coefficient, Lode parameter). The conditions of the analyses assumed constant values of *η* = 1 and *L* = 0.34. A high level of stress triaxiality was supposed to guarantee favourable conditions for void growth, while the value of the Lode parameter corresponded to a similar value found locally in similar void sheets [90]. For a system of dimensions (according to the diagram in Figure 8) *d*_1_ = 1, *d*_2_ = 1, a band of plastic localisation occurred between the two outer voids. Heterogeneous void growth was realised. Accelerated void growth leads to coalescence through an inner-neck mechanism between the inner void analysed and the outer voids. The internal void showed accelerated growth, with a 10% increase in volume at the time of coalescence with respect to the external voids. The change in the shape of the voids (high curvature zone) modifies the stress state through the analysed microstructure (notch effect) and accelerates the void growth process. The coalescence of the voids was defined using three parameters: the normalised void growth, the minimum distance between voids and the equivalent strain at the midpoint of the shortest path between adjacent voids. The coalescence is realised with a macroscopic equivalent strain of 0.11.

The evolution of the internal void position was analysed (when it takes a constant value of *d*_1_ = 1). With an increase in the value of the distribution of the internal void, there is an evolution of the void volume with an exponential character (for *d*_2_ = 1, the increase in the internal void at 1.95, for *d*_2_ = 4 an increase up to 7.8). Even for a large difference between the dimensions *d*_1_ and *d*_2_, the outer void shows a greater increase in void volume compared to the inner void. For a dimension *d*_1_ smaller than 4, coalescence through the inner necks is observed between pairs of voids: inner and outer. For *d*_2_ = 4, simultaneous coalescence is obtained between the three voids. The local strain just before the initiation of the coalescence process was determined to be in the range of 2.5–3.0. When the inner void moves away from the cluster of voids, the outer void grows more slowly. When only the presence of external voids is included in the model, coalescence occurs at a later stage, with a lower rate of void growth. For a constant value of the internal void position *d*_2_ = 1.75, an exponential increase in voids with a greater increase in the volume of internal voids than external voids were observed. For the external void position *d*_1_ = 3.25, no void coalescence occurred during the simulation. The level of effective deformation at the initiation of coalescence increases monotonically with the increasing dimension *d*_1_ (in the range 2.5–3.0) [89,91,92]. The significant role of void configurations in the formation of strain levels in the material was highlighted, among others, in [91], where the molecular dynamics simulation methodology was applied and, on this basis, two strain mechanisms were selected: local and homogeneous plastic strain. Local plastic strain is identified with necking and the occurrence of local shear. Similar results were presented in the work of Cadet et al. [92] where, for randomly distributed voids in the material, attention was paid to the destruction of the elementary cell in tension or by shear, which is relevant to the formation of the critical strain level at failure.

**Figure 9:**
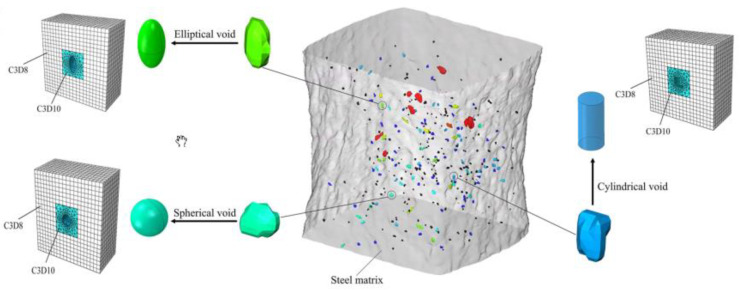
Shapes of voids in analysed steel were determined by computed tomography with developed numerical models, reprinted with permission from Ref. [94]. 2023, ASCE.

The shape of voids and their influence on the mechanism of void growth in the material has been the subject of many scientific studies [15,86,93,94,95,96,97,98,99,100,101]. In the paper [94], based on an in-situ observation of the material using high-quality μXCT computed tomography [3,102,103,104], different void shapes were selected, and in the next step, they were modelled in Abaqus using the RVE model. The initial shapes of the voids were analysed: cylindrical, spherical and elliptical, according to Figure 9. The relationships between the initial void shape and the nature of its growth were demonstrated in relation to the stress triaxiality, aspect ratio, orientation and initial void volume contribution. The lower the level of stress triaxiality, the greater the influence of the initial void shape on the void growth process. The volume of a spherical void increases the most, while the smallest increase is recorded for an elliptical void (at *η* = 0.33 in uniaxial tensile tests, determined through numerical calculations in the work of [105,106]). The higher the stress triaxiality level, the smaller the difference in the nature of void growth (to practically disappear when *η* = 2.0). The influence of the void shape factor on void growth occurs for stress triaxiality values below 0.8, in which case a cylindrical void shows greater growth than one with an elliptical shape (in proportion to the initial difference in shape factor). The initial void orientation—located in the direction opposite to the action of the tensile component—has the effect of increasing the voids more. The initial volume fraction had a smaller effect on the void growth than the other parameters analysed. On the basis of the observations made using computed tomography and numerical analysis, a formula was proposed to take into account the influence of the void shape on void growth at the mesoscale level [94]:

(2) $$\frac{f}{f_{0}}={\left(\frac{1.63}{\tilde{\eta }}\varepsilon_{eq}+1.0\right)}^{2.55{\tilde{\eta }}^{2.32}}\;\tilde{\eta }=\eta \left(1+{0.19e}^{-3.14\eta }\right)$$

where:

*f*—void volume fraction;

*f*_0_—initial void volume fraction;

η—stress triaxiality coefficient;

$\tilde{\eta }$—equivalent stress triaxiality considering void shape;

ε_*eq*_—equivalent strain.

An attempt to verify the classical theories relating to the phenomenon of the growth and coalescence of voids was realised in the work [107]. The considerations were carried out for copper rods, from which cylindrical specimens with circumferential notches of different radius values were made. The choice of the geometry of the test specimens allowed varying levels of stress triaxiality to be obtained. Two damage models were considered: the uncoupled Rice-Tracey model [108] and the coupled Gurson-Leblond-Perrin model [109]. Individual criteria relating to the void coalescence process were chosen for verification: the critical value of the damage parameter, the Brown and Embury criterion [110], the Thomason criterion [111] and a criterion based on the attainment of the maximum von Mises equivalent stress in a Gurson-type simulation [7]. Ellipsoidal voids were analysed, with the possibility of interaction between them. The influence of strain hardening could be taken into account by using the material (copper) after cold forming and in the annealed state. It was shown that the rate of void growth preceding the critical moment decreases with the level of stress triaxiality. A limiting value for the stress triaxiality coefficient within 1.5 was determined, where a change in the coalescence mode is likely to occur (void shape flattening, with consequent localisation of plastic strain). In verifying models relating to void coalescence, Thomason’s model described the mechanism well for the stress triaxiality range investigated (0.4–1.6). The Brown–Embury criterion was positively verified for the low triaxiality range (especially for low values of the strain hardening exponent), while the criterion gave worse results at high levels of triaxiality [22]. In the analyses involving a three-dimensional numerical model of a periodically hollow elastic-plastic solid, it was shown that increasing the initial void volume fraction (⨍) strongly influences the rate of void growth at high levels of triaxiality, with the porosity of the material as an additional determining factor [112]. At the moment of coalescence, the maximum value is reached by the von Mises effective stress [113,114]. In the paper [113], low-alloy steels containing inclusions of the manganese sulphide type were adopted for analysis. Two types of coalescence models were considered: those indicating the dominant role of localisation and the plastic boundary load model. Attention was paid to changes in the shape and spacing of the inclusions. On this basis, an attempt was made to obtain the data necessary for predicting the plastic fracture process. A cylindrical specimen model with axisymmetric notches was used in the FEM calculations.

### 3.2. Influence of the Stress State, Strain and Localisation

**Figure 10:**
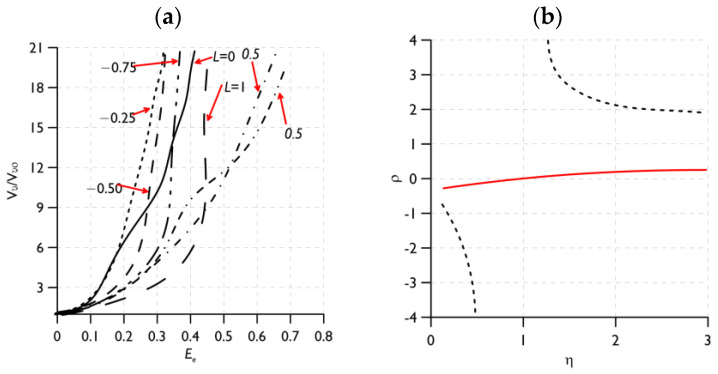
Results of the numerical analyses: (**a**) effect of the level of the Lode parameter on the growth of voids in the analysed material, adapted with permission from Ref. [118]. 2023, Elsevier, (**b**) relationship between the stress triaxiality coefficient and principal stress ratio (for different values of principal stresses), adapted with permission from Ref. [122]. 2023, Elsevier.

An important determinant of the growth and coalescence of voids is the prevailing state of stress [86,115]. A number of studies have attempted to assess the quantitative and qualitative evaluation of the influence of the most common stress triaxiality coefficient and the Lode parameter on the individual stages of ductile fracture realisation [116,117]. In the work [118], the influence of the Lode parameter on the realisation of the void mechanism in a material was determined. A micromechanical model of an elementary cell containing a spherically shaped void was used. Proportional loading was assumed with control of the stress triaxiality level and the Lode parameter. When analysing the change in the shape of the voids and their growth rate in the material, it was shown that the influence of the Lode parameter increases with the decreasing stress triaxiality level (Figure 10a). With the dominance of shear stresses, the localisation criterion in [119] cannot be considered as a measure of void coalescence. The construction of a micromechanical coalescence criterion is required. Attention has been drawn to the need to consider the void nucleation process when realising the final stage of destruction according to the ductile mechanism [120,121].

The influence of the level of stress triaxiality and the initial porosity of the material on the realisation of the mechanism—growth and void coalescence—was the subject of a paper by Kim and co-authors [122]. A representative material volume (RMV) model was used. A strong relationship was shown between the value of the stress triaxiality coefficient and the initial porosity of the material and the growth and coalescence of voids. A very important aspect of the analysis was highlighted: the stress triaxiality coefficient *η*, as an independent parameter, cannot be definitively associated with the initiation stage and nature of the course of void growth and coalescence. Figure 10b shows the relationship between the stress triaxiality (*η*) and principal stress (ρ) factors. The principal stress ratio is defined as the ratio of the highest to the lowest principal stress. There are cases where, for a selected level of stress triaxiality, there is a different macroscopic stress-strain response for void growth and coalescence (same value of principal stress ratio, Figure 10b) [122,123].

**Figure 11:**
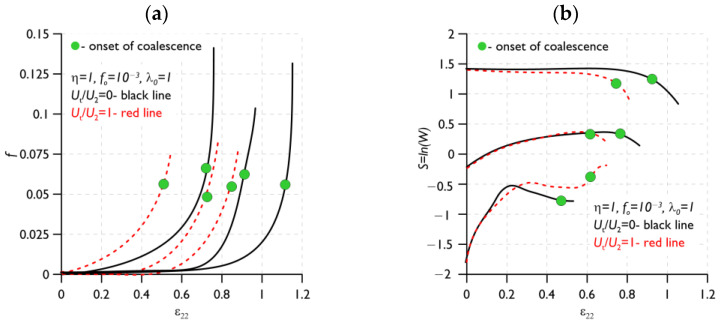
Results obtained using the elementary cell model, the relationship between: (**a**) porosity and axial strain, (**b**) void aspect ratio and axial strain, adapted with permission from Ref. [124]. 2023, Elsevier.

Several studies have attempted to determine the simultaneous influence of two or more variables—the stress triaxiality coefficient and the shear factor—on the course of the void mechanism in the material [124,125,126]. The paper [124] proposes an extension of the growth and coalescence model for ellipsoidal voids based on Gurson’s [7] proposal, taking into account the Gologanu [14] model for spheroidal voids. It was shown that the presence of shear, for the same levels of stress triaxiality, reduces the ductility. In the paper [125], the failure mechanism of primary voids in shear was analysed based on the results of numerical calculations. It was shown that the secondary particles rotate and elongate as a result of the reactions, up to a critical moment, associated with the occurrence of coalescence. Attention was drawn to the influence of the presence and interaction of secondary voids in the material on the coalescence process. The contribution of the shear component led to a reduction in the maximum stress value and accelerated the initiation of the void coalescence process. In particular, the contribution of the shear component is significant for large values of void form factors. A similar effect was obtained when analysing models with different void volume shares [127]. The plasticity increased with the increasing void aspect ratio values. The influence of the shear component is explained by analysing the magnitudes of the porosity evolution and the void form factor (Figure 11): flattened voids grow faster than elongated voids. Furthermore, elongated voids follow the rotation of the material. The contribution of the shear component causes the voids to grow faster, and thus the level of deformation needed to initiate coalescence decreases. Coalescence is controlled by the relative spacing between voids. The increase in ductility with a decrease in stress triaxiality stops at low stress triaxialities when the shear component is introduced into the model [120]. The decrease in ductility at low stress triaxiality is likely to be caused by the rotation of voids, which reduces the spacing between voids; in which case, favourable conditions exist for the realisation of coalescence.

The initial porosity has almost no effect on the rate of void rotation. It was shown that changes in the aspect ratio at the start of spherical voids are practically resistant to the contribution of the shear component to the interaction, while the aspect ratio is affected by the occurrence of void rotation. Due to the significant role of both the stress triaxiality and shear coefficient in the void coalescence process, a criterion based on the critical porosity of the material cannot be a reliable description of the coalescence process [124].

The simultaneous influence of three variables—the level of stress triaxiality, the Lode parameter and the value of shear stress—on the realisation of ductile failure was considered in the works of, e.g., [100,128,129,130]. The research in the selected papers was characterised by the use of micromechanical analysis. The focus was on the two final stages of the void mechanism: growth and void coalescence. Two coalescence mechanisms were distinguished: internal necking and at shear mode. With the development of shear bands (low triaxiality), shear localisation induces void coalescence in the material. For axisymmetric problems, the value of the Lode parameter has a significant effect on the orientation of the shear bands. The shear stress components cause the elongation and rotation of voids. This also influences the interaction between neighbouring voids, resulting in shear dominance during the coalescence process. An increased rate of void growth occurs when the ratio of principal normal stress to effective stress is increased for a given value of stress triaxiality and the Lode parameter. At medium and high levels of triaxiality, there is a faster stage of material cohesion loss associated with a faster rate of void growth. This is also reflected in lower effective strain levels at the onset of void coalescence. At low levels of triaxiality, despite applying shear stresses, the coalescence process is not determined [128]. Numerical calculations using the RVE model of the steel alloy (*E*/*σ*_YS_ = 300 and initial porosity *f*_0_ = 0.01) show that no coalescence of voids is observed when the following conditions are applied: Lode parameter (*L* = −1) and stress triaxiality coefficient *η* = 0.35. For parameters (*η* = −1, *L* = −1), (*L* = −0.99, *S* = 0.1225) and (*L* = −0.5, *S* = 0.8321), there was no uniaxial strain condition. Depending on the values of the Lode triaxial stress parameters and the shear ratio (*S*), void coalescence can be initiated without indicating the presence of a uniaxial strain state [118,130].

The significant role of the strain level on the mechanism of void growth and coalescence has been mentioned in a considerable number of scientific studies. Gao and Kim, in their paper [86], presented a failure criterion in the form of macroscopic equivalent strain (*ε*_c_) based on the configuration of the experimental results and numerical analyses of aluminium alloy 2024-T3. The equivalent strain was defined as a function of the stress triaxiality parameters and the Lode parameter. A FEM model of a representative material volume (RMV) was used. The impact of the initial shape and volume of the void and nucleation growth and the coalescence of secondary voids on the predicted damage area was considered. Attention was paid to the contribution of secondary voids to the growth and coalescence mechanism [86,131,132,133,134,135,136]. In the paper [131], the classical Gurson model was extended to take into account the effect of the void size in the material. It was shown that as the initial volume proportion of the voids increases, the effect of the void size on the course of ductile failure becomes more pronounced. The use of a non-local Gurson model enabled the size of the secondary voids in the matrix material to be described, with two populations of voids analysed [135]. It was assumed that the nucleation of secondary voids is controlled by the level of plastic strain, while it follows a normal distribution [137]. Secondary voids significantly accelerate the void coalescence process; however, at very low stress triaxialities, e.g., *η* = 0.33, coalescence cannot occur without their presence. It has been shown that the macroscopic equivalent strain *ε*_c_ decreases with the level of stress triaxiality, and the dependence of *ε*_c_ on *η* is more pronounced in the low triaxiality range. The equivalent strain increases with the Lode parameter (the highest sensitivity to a change in *L* was observed for the Lode range above 30°). The equivalent strain tends to increase in value with an increase in the aspect ratio of the primary void, while it decreases with the value of the initial volume fraction of the primary void [86].

The contribution of macroscopic strain localisation to the ductile failure mechanism has been highlighted in works including [23,85,138,139,140]. By causing a change in the uniform strain, localisation becomes the cause of damage in the material. Two mechanisms have been identified as the cause of macroscopic strain localisation in porous materials [85]:

Local degradation of the load-bearing capacity of the material. This occurs due to the softening of the material caused by microstructural changes (void growth mechanism) and progressive damage evolution. In turn, the degradation of the material’s load-bearing capacity causes the strain to be localised in a thin band. Void growth is the dominant mechanism resulting in macroscopic strain localisation. The initiation of the localisation process is determined by the material properties, the initial porosity, the orientation of the band undergoing localised strain and the stress state present [141].

Coalescence of voids. There is a local instability in which there is an interaction between voids in the material. The onset of coalescence is identified through the phenomenon of the concentration of plastic strain in the ligaments between adjacent voids, and this also causes changes in the nature of void growth. The level of stress triaxiality determines the cause of macroscopic localisation in the material [85].

The onset of localised strain is conditioned, among other things, by the sensitivity of the material to the strain rate [142,143,144,145]. An analysis of the effect of the strain rate on the two distinguished mechanisms of macroscopic strain localisation is presented in [145]. An elementary cell model containing initially spherical band voids was used, with the characteristics: *E* = 167 GPa, *σ*_YS_ = 418 MPa, *ε*_0_ = 0.0025, ν = 0.3, *N* = 0.1; with the material’s strain rate sensitivity parameter being *m* = 0–0.25. The strain triaxiality parameter ranged between 0.75–3, with a constant Lode parameter value of −1. It was shown that the level of critical equivalent strain in the band depends on the triaxiality value. For *η* < 2, the critical equivalent strains are the same at the onset of localisation, as well as coalescence, while the critical strains reach a higher value at the onset of coalescence for *η* > 2. For all values of the material’s sensitivity coefficient to the strain rate *m*, the critical equivalent strain at the onset of localisation and coalescence decreases with the increasing *η*. Analysing the magnitude of the critical void volume fraction *f*_c_, it was observed that the volume fraction is at a similar level at the onset of localisation and coalescence for low values of the stress triaxiality factor, so that for larger values of *η*, the volume fraction is larger at the onset of coalescence. For the entire range of *η* analysed, the critical volume fraction increases with the increase in parameter *m* [145].

In most cases, numerical analysis consists of considering an elementary cell containing a single void, most often of different geometries. This approach provided the analysis of the basic mechanisms involved in the growth and coalescence of voids in the material. It also resulted in a significant simplification of the numerical model, reducing the number of nodes and saving computational memory. In contrast, the interaction between voids and the randomness distribution of voids in the material was neglected. The necessity of a more accurate numerical model for describing the course of plastic failure in the material resulted in more studies on voids in the material, like two voids [132] or three voids [89,146,147,148]. In papers [92,149,150], an attempt was made to model the random distribution of voids in the material matrix in order to accurately describe the initiation and development of the void coalescence process.

**Figure 12:**
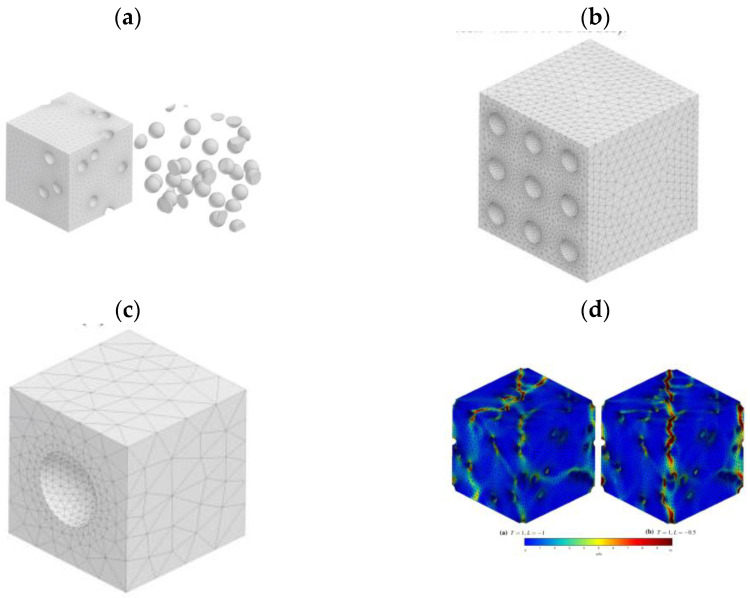
Computational models including a view of the finite element meshes: (**a**) random microstructure with 27 voids, (**b**) microstructure with 27 voids on a cubic lattice, (**c**) elementary cell with one void, (**d**) plastic strain levels and localisation paths for the model with 125 voids (after void coalescence), reprinted with permission from Ref. [92]. 2023, Springer Nature.

In [92], the numerical model assumed microstructures containing randomly distributed spherical voids in an elastoplastic matrix. In the numerical simulation program, a constant stress triaxiality and load parameter were assumed. The occurrence of large strains and the realisation of void coalescence were assumed. The selection of the initial size of the cell and the configuration of the voids in the matrix were chosen to ensure an initial porosity of the material of *f*_0_ = 6%. The distribution of defects (voids) was determined according to the Poisson sphere process [151]. A model with 27, 64 and 125 voids (for *η* = 1, *L* = −1 and *η* = 1, *L* = −0.5) was modelled to give a homogenised material (Figure 12a–c). Increasing the number of voids in the model significantly increased the calculation time and the load on the computational memory. When analysed with 125 voids, the results indicated the complex occurrence of localisation paths, but the main direction was parallel to or at 45° from the surface (Figure 12d) [150]. A very important aspect was the choice of damage indicator. In this case, the authors defined a damage index based on the loss of the full rank of the mean gradient rate of the strain component. This index provided a good estimate of the shear localisation in relation to others found in the literature. The modelled microstructures exhibited two competing failure mechanisms: tension and shear. The average critical stress level decreased with an increase in the number of voids in the model. This may be due to the higher probability of a favourable localisation path between the higher number of voids present [92].

**Figure 13:**
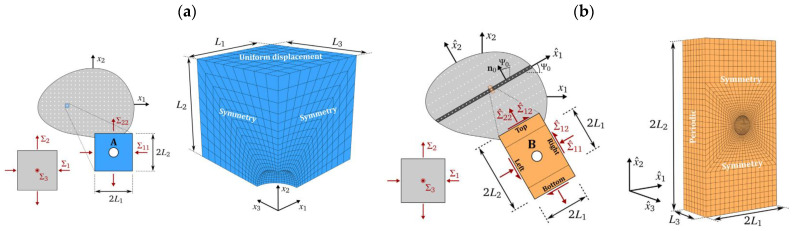

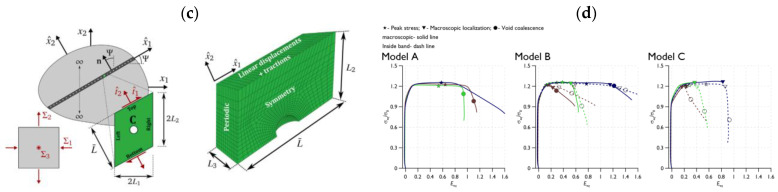
Three variants of the elementary cell were implemented for the analysis: (**a**) model *A*, (**b**) model *B*, (**c**) model *C*, (**d**) selected results of the analysis, reprinted with permission from Ref. [152]. 2023, Elsevier.

The impact failure initiation criterion and the macroscopic stress state include a significant effect on the realisation of the ductile fracture procedure. To verify the parameters mentioned in the earlier sections of the paper, three variants of the elementary cell were adopted for the analysis in the work [152], which assumed:

(a) An elementary cell with a single void when loaded with a normal component (Model *A*—Figure 13a);
(b) The model included finite-sized external blocks separated by a band, with both normal and shear load (imperfection bands) (Model *B*—Figure 13b) [153];
(c) The model had semi-infinite external blocks, assuming loading through normal and shear components (imperfection bands) (Model *C*—Figure 13c) [153].

In each case, the base material in the numerical model was an aluminium alloy. As expected, the assumptions of the computational model determined the mechanical response and the level of critical strain. If the point of failure occurring in the model is related to the value of the Lode parameter, it is shown that only in the case of model *A* was there a monotonic increase in the level of critical strain with a change in the value of the Lode parameter. The differences in the performance of the models at the critical strain level disappear with the adoption of a constant value of the Lode parameter. It was shown that for the analysed models, in most cases of strain at the maximum strain level, the localisation and coalescence of voids are different. In the models labelled *B* and *C*, if the band values are considered, localisation usually occurs between the occurrence of the maximum stress level and the onset of void coalescence. For model *C*, if an intermediate value of stress triaxiality is assumed, the onset of the localisation and void coalescence processes coincide. The individual relationships for the analyses carried out are shown in Figure 13d. As the triaxiality increases, localisation will precede the void coalescence process. In all cases, it is observed that the stress state, the selection of a computational model and the reference assumptions had a significant influence on the results [85,152,154,155].

### 3.3. Parameter Analysis in Front of the Crack Tip

**Figure 14:**
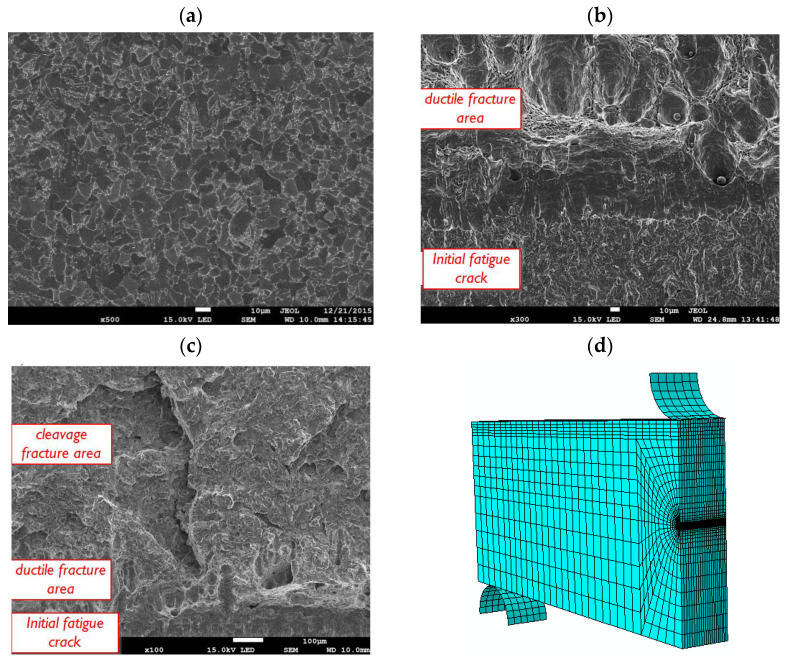
Material (S355 steel) and test specimen: (**a**) microstructure of the steel [156], (**b**) ductile fracture (test temperature +20 °C) [156], (**c**) mixed cleavage-ductile fracture (test temp. −80 °C) [158], (**d**) numerical model of the SENB specimen in Abaqus, reprinted with permission from Ref. [159]. 2023, Elsevier.

In the papers [156,157] and in our own research, we analysed the fracture mechanisms occurring in S355 steel of a ferrite microstructure with coagulated carbide particles (Figure 14a) (strength characteristics at room temperature +20 °C: yield strength: *σ*_YS_ = 412 MPa, ultimate tensile strength *σ*_UTS_ = 602 MPa, relative elongation *A*_5_ = 29%). The research involved the determination of the basic strength and ductility characteristics in uniaxial tensile testing, as well as the fracture toughness characteristics. Laboratory tests were carried out over a wide temperature range, between +20 °C and −120 °C. A ductile fracture realised according to the mechanism of initiation, growth and void coalescence was recorded in the case of mixed, cleavage and ductile crack development (Figure 14c). In this case, the ductile fracture area occurred between the crack front and the cleavage fracture area. An increase in the test temperature led to an increase in the proportion of ductile fracture, up to the complete dominance of the fracture according to the ductile mechanism—through the void development mechanism (Figure 14b).

**Figure 15:**
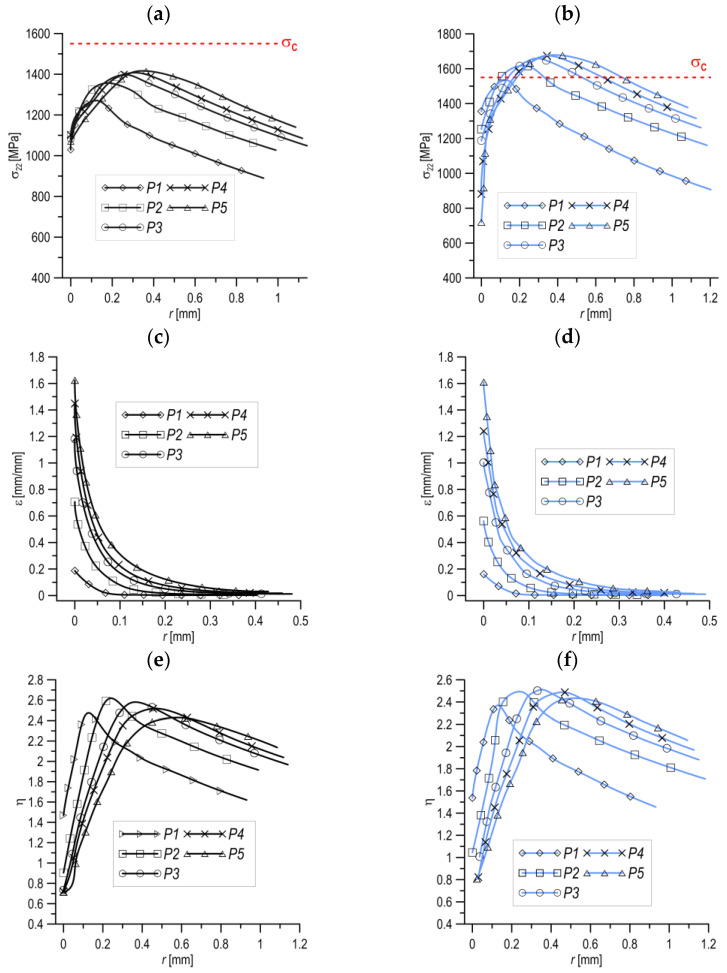
Numerically determined value distributions in front of the crack tip, SENB specimen, steel S355: opening stresses: (**a**) +20 °C, (**b**) −80 °C, plastic strain: (**c**) +20 °C, (**d**) −80 °C, stress triaxiality coefficient: (**e**) +20 °C, (**f**) −80 °C.

A model of a three-point bending SENB specimen was adopted for the numerical calculation programme, with dimensions identical to those used for the experimental fracture toughness tests (Figure 14d). Several points on the force-displacement diagram recorded during the fracture toughness tests were selected. These points were used to load the SENB specimen by applying displacement to the top roll in a numerical simulation (for +20 °C: P1 = 0.45 mm, P2 = 0.80 mm, P3 = 1.10 mm, P4 = 1.30 mm, P5 = 1.60 mm, for temp. −80 °C: P1 = 0.41 mm, P2 = 0.80 mm, P3 = 1.00 mm, P4 = 1.20 mm, P5 = 1.45 mm). The purpose of selecting several displacement values for a given specimen was to determine the nature of the formation and development of the mechanical fields in front of the crack front. As a result of the numerical calculations, the distributions of stress, strain and the stress triaxiality parameter in front of the crack front were determined, shown in Figure 15.

An increase in the test temperature leads to an increase in the level of strain in front of the crack front, while the value of the stress triaxiality factor *η* decreases. Based on the numerical calculations, it was established that the realisation of the fracture according to the mechanism of the initiation, growth and coalescence of voids is possible in the case of the presence of a high level of plastic strain and relatively high values of the stress triaxiality factor. In the cases studied, for S355 steel: *ε* ≈ 1.2–1.6 and *η* ≈ 0.72–1.2. Based on the results obtained, it can be concluded that the following conditions are necessary for the realisation of ductile fracture: the occurrence of high levels of strain *ε* and the achievement of a relatively high level of stress triaxiality *η* [156,157,160].

### 3.4. Mesoscale Modeling, Linking Microstructure and Macroscopic Properties

As mentioned in the introduction, the recognition of microstructural phenomena should be the basis for the formulation of material models that allow for the description of the material behaviour on larger scales (meso and macro), as well as in relation to elements on engineering scales. Usually, due to the limitation of the computational cost, it is reasonable to apply the porous material only directly in the process zone, while in the remaining area, it is sufficient to use a simple model of the elastic-plastic material (without damage).

**Figure 16:**
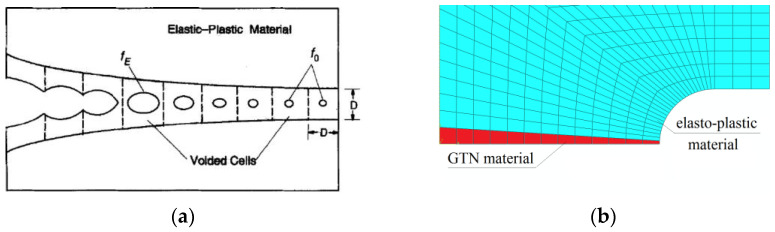
Cellular model of a porous material (**a**) Concept, reprinted with permission from Ref. [161]. 2023, Elsevier, (**b**) An example of application for modelling notched specimens [29].

This approach was described by Xia and Shih [161], who proposed to place elementary cells with an internal void in the process zones. The influence of the cavity on the overall material behaviour is captured using a porous material model (for example, the Gurson-Tvergaard-Needleman model, mentioned before). This concept is illustrated in Figure 16a.

An example of the application of the cellular model to simulate the ductile failure of notched specimens made of S355 steel is described in [29]. Due to the symmetry of the geometry and load, an axisymmetric model of half of the tensile element was developed (Figure 16b). The GTN porous material model was applied at the notch bottom, i.e., in the area of expected failure. Importantly, the parameters of the material model were determined experimentally (the volumetric fraction of voids at various stages of loading was determined by microscopic observations), while the parameters of void nucleation were determined numerically, as presented in Figure 2.

**Figure 17:**
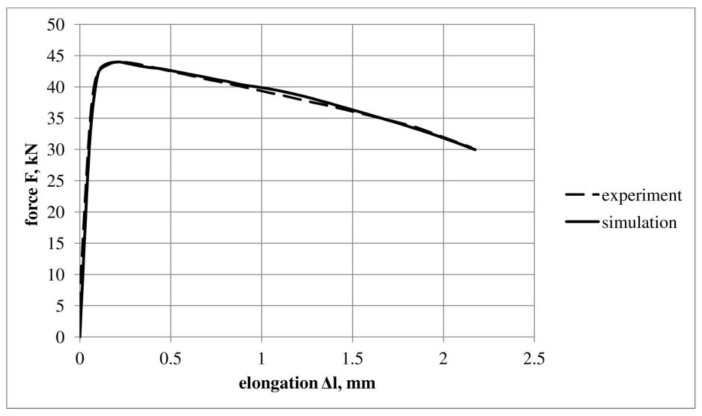
Comparison of the results of the experiment and simulation of tensile tests of notched elements, modelling carried out using the GTN model and experimentally determined microstructural parameters of S355 steel [29].

Figure 17 presents the comparison of the experiment and simulation results (force-displacement curves). The use of the porous material model and its experimentally/numerically determined parameters results in good agreement between the simulation and experiment, also on the macroscopic scale.

## 4. Summary, Conclusions and Suggestions for Future Work

The use of numerical simulations in solving problems related to the description of failure according to the ductile mechanism is part of the trend toward a local approach to analysis. The local, most stressed area in the material is then considered. Supplementing analytical criteria, in-situ observations and the results of numerical calculations allow a hybrid approach to the analysis of the void mechanism in the material. In most literature studies, the basis for numerical calculations is the use of an elementary cell model. This can contain one or more modelled voids, with different shapes and proportions of void contribution to the matrix (material). Calculations using the elementary cell model make it possible to determine the overall response of the material in terms of a selected physical property, with an appropriate definition of the boundary conditions. This paper identifies solutions to include more voids with random distributions in the numerical model, and their influence on the process of void growth and coalescence in the material. The location of the fracture and the nature of the course of the growth and coalescence mechanisms are very sensitive to the spatial distribution of voids. A determinant influence on the realisation of the void mechanism in the material is the existing stress state. This can be defined by the magnitude of the stress triaxiality factor or the Lode parameter (or angle). In the selected studies in this paper, the numerical model considers the stress and the simultaneous effect of the shear coefficient on changes in the shape and distribution of voids in the material. An important aspect is to consider strain localisation, causing a change from constant strain and the destruction of the material. This is reflected in two phenomena in the material: the local degradation of the load-bearing capacity and the void coalescence mechanism as the final stage of the void mechanism. Numerical analyses of materials with a ductile failure mode according to the void mechanism can be used to determine the size before the crack tip in elements with cracks and can, on this basis, formulate the failure criteria.

Despite decades of research, there are still many aspects of void development that have not been fully explained, either experimentally or numerically. To date, little is known about the development of voids under low triaxiality conditions, where the Lode angle/parameter is of decisive importance. Another phenomenon to be investigated in the future is the development of flattened voids, as well as the effect of void locking on its growth.

Numerical simulations of void nucleation and development currently mostly focus on 2D or periodic 3D structures. A comprehensive approach in which real spatial structures are analysed—captured, for example, by means of microtomography—is very rare at present. Moreover, little attention is paid to the statistical nature of void development.

At present, few studies have been published on the influence of parameters such as the strain rate or temperature on void development. These are issues of great practical importance, as they enable the description of the material behaviour in emergency situations, for example, in fire conditions.

From the point of view of the engineering application of material models taking into account the growth of voids, a significant problem is the lack of standardised void nucleation and growth parameters for typical materials (e.g., steel) used in engineering structures.

The ‘bottom-up’ approach offers great potential in understanding the mechanics of damaged materials, which uses models known from chemistry and physics to link the properties, at the atomic level, with the macroscopic parameters of the material. However, due to the high complexity of the problem and today’s computing power limitations, this solution is currently difficult to implement.

## Data Availability

No new data were created or analysed in this study.
